# Vaginal Probiotic Potential of *Lactobacillus acidophilus*: Population Genomic and Phenotypic Analysis

**DOI:** 10.3390/genes17091009

**Published:** 2026-08-26

**Authors:** Yixin Mao, Yanqing Che, Mengjie Li, Guodong Yan, Xiao Liu, Ruocheng Yang, Hongzhou Li, Yeshun Fan, Haojie Zhan, Zhiwen Sun, Xuemei Bai, He Gao, Duochun Wang

**Affiliations:** 1 National Key Laboratory of Intelligent Tracking and Forecasting for Infectious Diseases, National Institute for Communicable Disease Control and Prevention, Chinese Center for Disease Control and Prevention & Chinese Academy of Preventive Medicine, Beijing 102206, China; maoyixin@nieh.chinacdc.cn (Y.M.); cheyanqing99@gmail.com (Y.C.); mjlee97@163.com (M.L.); yanguodong0208@163.com (G.Y.); liuxiao930919@163.com (X.L.); yrc15270369345@163.com (R.Y.); hongzhou379@gmail.com (H.L.); fanyeshun2000@163.com (Y.F.); 18602782325@163.com (H.Z.); sunzhiwen@icdc.cn (Z.S.); baixuemei@icdc.cn (X.B.); gaohe@icdc.cn (H.G.); 2 National Key Laboratory of Intelligent Tracking and Forecasting for Infectious Diseases, National Institute of Environmental Health, Chinese Center for Disease Control and Prevention & Chinese Academy of Preventive Medicine, Beijing 100021, China; 3 China CDC Key Laboratory of Environment and Population Health, National Institute of Environmental Health, Chinese Center for Disease Control and Prevention & Chinese Academy of Preventive Medicine, Beijing 100021, China; 4 School of Public Health, Lanzhou University, Lanzhou 730000, China; 5 School of Public Health, Gansu University of Chinese Medicine, Lanzhou 730000, China

**Keywords:** *Lactobacillus acidophilus*, vaginal probiotic, pan-genome, vaginal microbiota, antimicrobial activity, reproductive tract infection

## Abstract

**Background/Objectives**: Certain strains of *Lactobacillus acidophilus* are widely used as probiotics. However, their functional potential for female reproductive tract health remains insufficiently characterized. While most studies have focused on individual strains, the distribution of putative probiotic-associated genes across the species remains unclear. This study aimed to evaluate the vaginal probiotic potential of *Lb. acidophilus* using population genomic analysis and comparative phenotypic characterization. **Methods**: Pan-genomic analysis was performed on 109 *Lb. acidophilus* genomes (107 public genomes and 2 vaginal isolates). Putative probiotic-associated gene clusters were identified by functional annotation, categorized into functional modules, and compared among ecological-origin groups. Two vaginal isolates (strains A2 and A3) were characterized *in vitro* for growth under different pH conditions, cell surface hydrophobicity, lactic acid and hydrogen peroxide production, antimicrobial activity, hemolysis, and antimicrobial susceptibility. **Results**: The *Lb. acidophilus* pan-genome was closed, with 1782 of 1902 gene clusters (93.69%) classified as core. Thirty-six putative probiotic-associated gene clusters were identified and grouped into four modules: environmental tolerance, adhesion/colonization, exopolysaccharide/biofilm synthesis, and nutrient metabolism/microbial competition. Thirty-four of the 36 gene clusters were present in all 109 genomes, and no general ecological origin-specific distribution pattern was observed. Three bacteriocin-related gene clusters were conserved across all genomes. A2 and A3 exhibited similar lactic acid production and growth patterns at pH 4–6. Both produced relatively low amounts of hydrogen peroxide compared with the reference strains. A3 showed higher cell surface hydrophobicity and moderate inhibition against *Gardnerella vaginalis*, while A2 showed no inhibition of this organism. Both isolates were non-hemolytic, and were susceptible to vancomycin and linezolid, resistant to clindamycin, and non-susceptible to daptomycin. No acquired antibiotic resistance genes were detected. **Conclusions**: Most putative probiotic-associated gene clusters were conserved across the *Lb. acidophilus* population, whereas A2 and A3 showed strain-dependent phenotypic differences. These findings support combining population genomic analysis with strain-level phenotypic testing when selecting *Lb. acidophilus* candidates for bacterial vaginal infections.

## 1. Introduction

Reproductive tract infections (RTIs), such as bacterial vaginosis (BV) and vulvovaginal candidiasis (VVC), are common gynecological diseases with extremely high prevalence worldwide. Their occurrence and progression are closely associated with dysbiosis of the vaginal microbiota [1,2]. The primary etiological agents of BV are bacterial pathogens, such as *G. vaginalis*. In many healthy women, *Lactobacillus* species are predominant members of the vaginal microbiota. They inhibit the adhesion, colonization, and proliferation of pathogens through various mechanisms, including competitive adhesion, production of acidic substances (maintaining a low pH environment), secretion of antimicrobial compounds (e.g., bacteriocins), and modulation of epithelial cell responses, thereby maintaining female vaginal micro-ecological health [2,3]. However, conventional antimicrobial therapy remains the mainstay of treatment for RTIs; for bacterial conditions such as BV, metronidazole and clindamycin are commonly used. Recurrence after treatment remains a clinical challenge, and antimicrobial resistance may further complicate long-term management. Probiotic therapy, which aims to restore and enhance the intrinsic protective microbiota of the female reproductive tract, has therefore attracted increasing interest as a potential alternative or adjunctive approach to conventional treatment. Several studies have investigated the potential of certain lactic acid bacteria (LAB) for managing RTI-related conditions [4,5]. However, compared with the well-developed field of intestinal probiotics, the development of probiotics specifically targeting the female reproductive tract is lagging, with inconsistent clinical outcomes [6,7].

*L. acidophilus* was first isolated from infant fecal samples [8,9]. Some *Lb. acidophilus* strains have been reported to tolerate acid and bile salts, and exert multiple beneficial effects on the human body, including nutritional effects, regulation of intestinal microbiota balance, enhancement of immunity, anti-aging and anti-cancer properties, and support of cholesterol reduction [8]. In recent years, research on the probiotic functions of *Lb. acidophilus* has primarily focused on metabolic diseases, anti-tumor effects, and psychiatric disorders [10,11,12,13,14]. Some studies have suggested that certain strains (e.g., La5, NCFM) have the potential to inhibit oral and urogenital pathogens (e.g., *Porphyromonas gingivalis*, *Tannerella forsythia*, *Klebsiella pneumoniae*, and *Pseudomonas aeruginosa*) [15,16]. In the context of female reproductive tract health, few *Lb. acidophilus* strains have been isolated from the vagina. Some have been reported to inhibit *Candida albicans* [17]. However, oral administration of a probiotic formulation containing strains, including *Lb. acidophilus*, *Lacticaseibacillus paracasei*, *Lacticaseibacillus rhamnosus*, *Bifidobacterium bifidum*, and *Bifidobacterium lactis*, failed to significantly reduce vulvovaginal infections in pregnant women, and the vaginal colonization of administered strains was not detected [18]. These findings highlight critical gaps in the current research on *Lb. acidophilus* for reproductive tract applications: the lack of systematic validation linking genomic characteristics to phenotypic outcomes, unclear compatibility of strain origin with the vaginal microenvironment, and evidence suggesting that orally administered strains not originating from the reproductive tract struggle to establish vaginal colonization. Together, these observations underscore the necessity of selecting vaginal-derived strains with both genetic suitability and phenotypic advantages.

Based on the above background, this study aims to evaluate the potential of *Lb. acidophilus* for applications in female reproductive tract health through a systematic strategy from population to individual, from genotype to phenotype. First, pan-genome analysis of the *Lb. acidophilus* population was performed to elucidate its core genomic features, with a focus on identifying gene sets associated with vaginal adaptation and probiotic functions. The population genomic analysis was designed to assess the prevalence and conservation of candidate genes potentially associated with vaginal probiotic functions across available *Lb. acidophilus* genomes. Phenotypic assays were subsequently performed on two vaginal isolates, A2 and A3, to compare probiotic-relevant phenotypes and support strain-level prioritization. This experimental component was intended as proof-of-concept strain characterization. This study aims to provide a candidate probiotic species with a well-defined genomic background and experimental evidence for the prevention and treatment of female RTIs, thereby laying a foundation for subsequent mechanistic studies and clinical development.

## 2. Materials and Methods

### 2.1. Bacterial Strains

*Lb. acidophilus* A2 and A3, *Lactobacillus crispatus* VJ31, and *Lactobacillus gasseri* VL3 were isolated from reproductive tract secretion samples collected from healthy women. *Lactobacillus crispatus* CGMCC 1.2743, *Lactobacillus delbrueckii* subsp. *bulgaricus* GDMCC 1.2783, and *Lactobacillus jensenii* GDMCC 1.1309 were obtained from CGMCC or GDMCC. *Lb. delbrueckii* subsp. *lactis* DM8909, originally isolated from a clinical medicine used for BV treatment, was included as a positive control. All strains were identified by MALDI-TOF MS, and their 16S rDNA was extracted, sequenced, and compared against the NCBI database for identification or confirmation. All *Lactobacillus* strains and pathogenic strains are currently maintained at the Center for Human Pathogenic Culture Collection of the Chinese Center for Disease Control and Prevention (CDC). Pathogenic bacteria include *G. vaginalis* (G.V.), *Streptococcus agalactiae*, *Staphylococcus aureus*, and *Escherichia coli*, with more detailed information provided in Table S1. Two *Lb. acidophilus* isolates, A2 and A3, were subjected to the same phenotypic assays, including growth under different pH conditions, cell surface hydrophobicity, lactic acid and H_2_O_2_ production, antimicrobial activity, and safety assessment.

### 2.2. Genome Sequencing, Assembly and Annotation

Publicly available genome assemblies of *Lb. acidophilus* were downloaded from the NCBI database in September 2024. Following initial assembly filtering, genome quality was assessed using QUAST v5.3.0 [19]. Assemblies with ≤200 contigs and CheckM contamination of ≤1% were retained. The final dataset comprised 107 publicly available genomes and two vaginal isolates sequenced in this study, A2 and A3, yielding a total of 109 genomes. Accession numbers and available metadata for all genomes, including collection date, geographic origin, and isolation source, are provided in Supplementary Table S2. Lab-isolated strains underwent genomic DNA extraction using the FastPure Bacteria DNA Isolation Mini Kit (Vazyme, Nanjing, China) and whole-genome sequencing on the Illumina platform by Shanghai Majorbio Bio-pharm Technology Co., Ltd. (Shanghai, China). Following raw data quality control, statistical analysis, and genome evaluation, *de novo* assembly was performed on clean data using short-read assembler SOAPdenovo v2.04 [20]. Plasmid replicons were screened using PlasmidFinder v2.1.6 [21]. Plasmid sequences were compared against the PLSDB database [22] (https://ccb-microbe.cs.uni-saarland.de/plsdb/, accessed on 3 April 2025) using BLASTn v2.12.0 (https://blast.ncbi.nlm.nih.gov/Blast.cgi, accessed on 3 April 2025). Coding sequences (CDS) in single-strain genome sequences were predicted using Prodigal v2.6.3 [23]. tRNA prediction was performed using tRNAscan-SE v2.0 [24] (http://trna.ucsc.edu/software/, accessed on 8 April 2025), and rRNA prediction was performed using Barrnap v0.9 (https://github.com/tseemann/barrnap, accessed on 8 April 2025). Other small non-coding RNA genes (sRNAs) were identified using Infernal v1.1.5 [25] (http://eddylab.org/infernal/, accessed on 8 April 2025) against the Rfam database [26] (https://rfam.org/, accessed on 8 April 2025).

### 2.3. Average Nucleotide Identity (ANI), Pan-Genome and Phylogenetic Analysis

Genome annotation was performed using Prokka v1.14.6 [27] for all genomes. ANI was calculated using fastANI v1.34 [28] to assess species delineation among all genomes. Pan-genome analysis was conducted using Panaroo v1.5.2 [29] in strict mode with the annotated GFF files. For phylogenetic analysis, core genome SNP alignment was generated using Snippy v4.6.0 (https://github.com/tseemann/snippy, accessed on 30 May 2025) with GCA_000389675.2 as the reference genome. Recombination regions were detected and removed using Gubbins v3.4 [30]. A maximum likelihood phylogenetic tree was inferred using FastTree v2.1.11 [31] under the generalized time-reversible (GTR) model with a CAT approximation for rate heterogeneity. The phylogenetic tree was visualized and annotated using the online tool iTol [32] (https://itol.embl.de/, accessed on 30 March 2026).

### 2.4. Functional Annotation and Identification of Putative Probiotic-Associated Genes

Protein-coding genes from all 109 genomes were functionally annotated using eggNOG-mapper v.2.1.12 [33]. Annotation entries with an e-value < 1 × 10^−5^ were retained. A broad keyword-based search was then conducted to identify candidate gene clusters related to reproductive tract probiotic functions. Candidate entries were individually reviewed based on their eggNOG functional descriptions and associated KEGG, COG, and GO annotations to exclude nonspecific or functionally unrelated hits. The query IDs of the retained entries were subsequently mapped to Panaroo gene cluster IDs and the gene presence–absence matrix. Apparent absences of core gene clusters were re-examined by searching their aligned sequences against the corresponding genome assemblies using BLASTn. The local organization of the identified sequences was subsequently compared with that of the two closest gene-positive genomes selected from the core SNP phylogeny.

Based on available isolation metadata, genomes were grouped by ecological origin as vagina, intestine, human (with an unspecified anatomical source), food, animal, or unknown. The prevalence of each putative probiotic-associated gene cluster was calculated as the proportion of genomes carrying that gene within each group.

Putative biosynthetic gene clusters (BGCs) and ribosomally synthesized and post-translationally modified peptides (Ripps) in *Lb. acidophilus* A3 were predicted using BAGEL4 [34] (http://bagel4.molgenrug.nl/, accessed on 26 December 2025) and antiSMASH v8.0.4 [35] (https://antismash.secondarymetabolites.org/, accessed on 26 December 2025).

### 2.5. Environmental Tolerance: Growth Ability Under Different pH Conditions

MRS broth (Oxoid, Basingstoke, UK) media with varying pH values (3, 4, 5, 6, 7) were prepared. Strains were inoculated into MRS broth (pH 4.8–5.0) for activation and incubated statically at 37 °C with 5% CO_2_ for 20–24 h. Suspensions were adjusted to 0.5 McFarland standards, corresponding to an approximate bacterial concentration of 1.5 × 10^8^ CFU/mL. The bacterial suspensions were then inoculated at a 1% inoculum size (*v*/*v*) into MRS broth media with different pH values and incubated statically at 37 °C with 5% CO_2_ for 18–20 h. OD_600_ was measured to evaluate the growth of strains under different pH conditions.

### 2.6. Adhesion Activity: Cell Surface Hydrophobicity Assay

Cell surface hydrophobicity was determined using the microbial adhesion to hydrocarbons (MATH) method [36]. Bacterial cells were harvested by centrifugation at 4000 × g for 15 min, washed three times with PBS (Solarbio, Beijing, China), and resuspended in PBS to an optical density of 0.8 at 600 nm (OD_600_). Three milliliter aliquots of the bacterial suspension were mixed with either xylene or hexadecane (both Maikelin, Shanghai, China). A control group without hydrocarbon was included. The mixtures were vortexed for 1 min and allowed to stand for 15 min to achieve phase separation. The aqueous phase was carefully collected, and its OD_600_ was measured using PBS as a blank. Three replicates were performed for each strain. The hydrophobicity rate was calculated using Formula (1):


(1) $$Hydrophobicity\left(\%\right)=\frac{{OD}_{600}\;of\;control-{OD}_{600}\;of\;experimental}{{OD}_{600}\;of\;control}\times 100$$

### 2.7. Metabolic Activity: Determination of Lactic Acid Production

Lactic acid production was measured using a modified method based on AOAC 947.05 [37] and Jiang et al. [38]. Bacterial culture was incubated at 37 °C for 66 h, with a blank medium control. The volume of 0.1 M sodium hydroxide (NaOH) standard solution titrant consumed was calculated as formula (2):

(2) $$X=(V_{1}-V_{2})\times C_{NaOH}\times 0.09\times 100/V_{s}$$

where X = acid production of the sample (g/100 mL); V_1_ = volume of NaOH consumed by the sample (mL); V_2_ = volume of NaOH consumed by the blank medium (mL); C_NaOH_ = concentration of NaOH solution (mol/L); 0.09 = conversion factor for lactic acid; V_s_ = volume of the sample solution (mL).

### 2.8. Metabolic Activity: Determination of Hydrogen Peroxide (H_2_O_2_) Production

H_2_O_2_ production was measured using a modified method based on Kang et al. [39] and Martín et al. [40]. Bacterial cells were harvested by centrifuging at 12,000× *g* for 20 min at 4 °C, washed with 50 mM PPB (pH 6.0; Innochem, Beijing, China), and resuspended in PPB with 5 mM glucose. Mixture was then diluted 20-fold with PPB containing glucose and incubated aerobically at 37 °C for 24 h. Five milliliters of the supernatant was mixed with 1 mL horseradish peroxidase solution (Solarbio, Beijing, China) and 0.1 mL tetramethylbenzidine (Maikelin, Shanghai, China). The tubes were incubated at 37 °C for 10 min, and 0.2 mL of 4 N HCl was added to stop the reaction. The H_2_O_2_ concentration was determined by comparing OD_400_ values with the standard curve generated under the same conditions [41]. Briefly, standard solutions of H_2_O_2_ were prepared in acetate buffer at concentrations of 1, 2, 3, 4, and 5 μg per mL. Each standard solution was further diluted, and the absorbance of each standard was measured at 400 nm. A standard curve was constructed by plotting the optical density readings against the amount of H_2_O_2_ (μg).

### 2.9. Microbial Competition: Antimicrobial Activity Against Pathogens

The antimicrobial activity was determined using a modified spot-on-lawn method [42]. *Lactobacillus* strains were cultured in MRS medium at 37 °C with 5% CO_2_ for 20–24 h, and the concentration was adjusted to approximately 1 × 10^8^ CFU/mL. A 2 μL suspension was spotted onto the surface of MRS medium containing 1.5% (*w*/*v*) agar and incubated at 37 °C with 5% CO_2_ for 24 h. Pathogenic strains (Table S1) were recovered, transferred to appropriate media, adjusted to a concentration of 1 × 10^8^ CFU/mL, and incubated for 24 h. A 1 mL pathogen suspension was mixed with 100 mL of agar-containing liquid medium and poured onto the pre-spotted plates with *Lactobacillus* strains. Plates were incubated for 48 h. The diameters of inhibition zones were measured (mm). The criteria for evaluation were as follows: inhibition zone < 7 mm, 7–12 mm, or >12 mm, were considered negative, moderate, or high inhibition, respectively.

### 2.10. Safety Evaluation

#### 2.10.1. Virulence Factor (VF) Genes and Antibiotic Resistance Genes (ARGs)

Virulence genes were annotated against VFDB [43] (the Virulence Factor Database, https://www.mgc.ac.cn/VFs/, accessed on 16 April 2025) using a threshold of ≥80% identity and ≥70% coverage. ARGs were annotated against CARD [44] (the Comprehensive Antibiotic Resistance Database, https://card.mcmaster.ca, accessed on 16 April 2025) with a threshold of ≥95% identity and ≥90% coverage.

#### 2.10.2. Antibiotic Resistance Phenotype

Antimicrobial susceptibility testing (AST) was performed based on MIC using custom plates (Thermo Fisher Scientific, Waltham, MA, USA) coated with multiple antibiotics (azithromycin, amikacin, trimethoprim sulfamethoxazole, vancomycin, rifampin, gentamicin, linezolid, clindamycin, oxacillin + 2% NaCl, nitrofurantoin, daptomycin, and florfenicol). AST results were interpreted according to the Clinical and Laboratory Standards Institute (CLSI) M45 Ed3 (2016)’s guidelines. Quality control strains were *E. coli* ATCC 25922 (Table S1).

#### 2.10.3. Hemolysis

Strains were recovered in MRS medium for 20–24 h and adjusted to a concentration of approximately 1 × 10^8^ CFU/mL. A 5 μL aliquot was spotted onto blood agar plates (Oxoid, Basingstoke, UK) and incubated anaerobically at 37 °C. Hemolysis was monitored every 24 h to determine the hemolytic type (α, β, or γ).

## 3. Results

### 3.1. ANI, Pan-Genome and Evolutionary Characteristics of Lb. acidophilus Population

**Figure 1:**
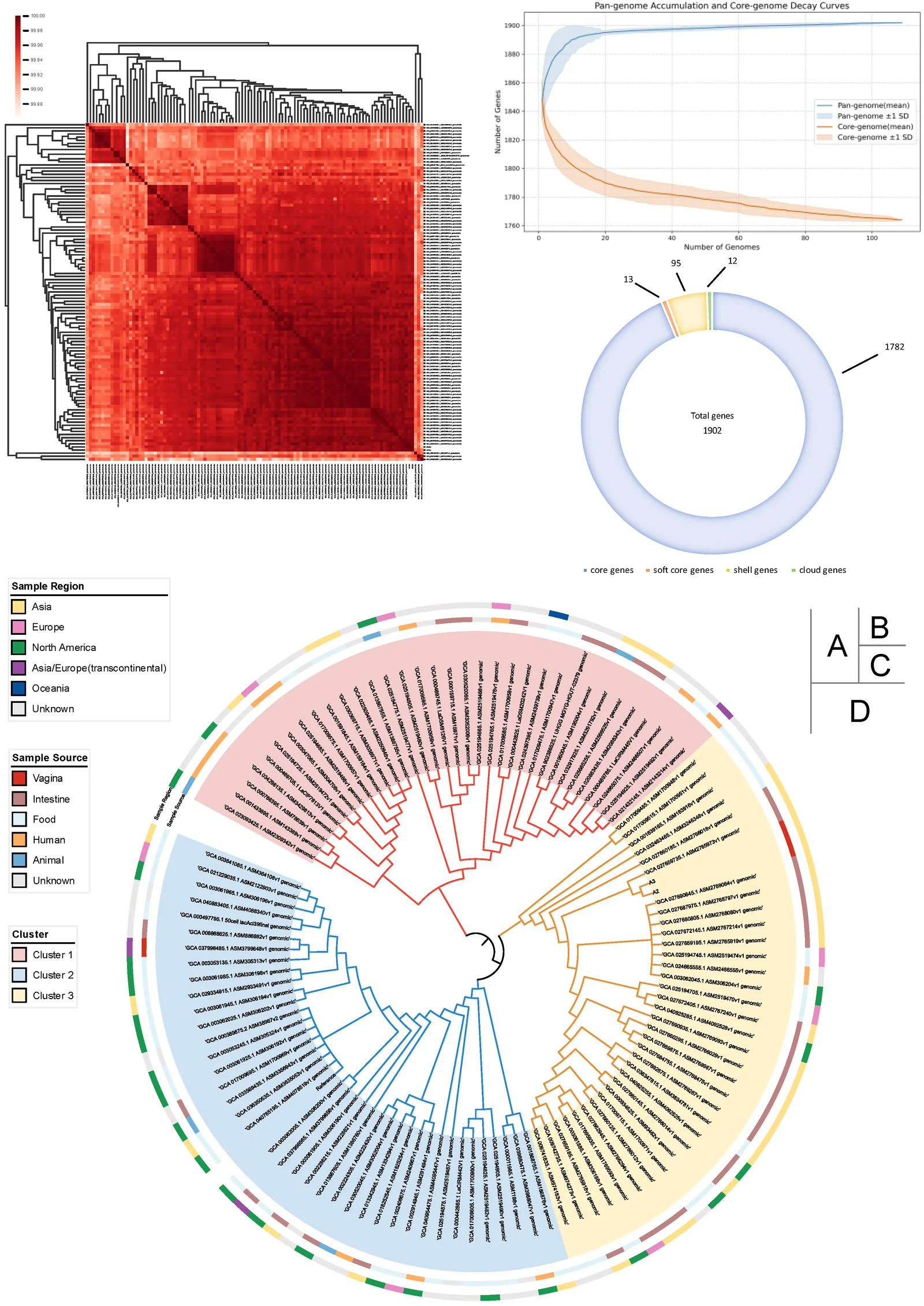
Genomic features of *Lb. acidophilus*. (**A**) Pairwise ANI heatmap with an average linkage hierarchical clustering dendrogram based on transformed ANI dissimilarities. (**B**) Pan-genome accumulation and core genome decay curves with error bands. (**C**) Total gene count. (**D**) Circular representation of the recombination-filtered core SNP maximum-likelihood phylogenetic tree.

The average genome size of *Lb. acidophilus* is 1.97 Mb. All strains exhibited ANI values above 99%, indicating they are the same species (Figure 1A). As the number of sequenced strains increased, the rate of discovery of new genes gradually decreased and leveled off (Figure 1B). Heaps’ law fitting yielded a γ value of 0.0037 (95% CI: 0.0034–0.0039; R^2^ = 0.8612), indicating a closed pan-genome and suggesting that the gene pool of *Lb. acidophilus* is largely saturated by the currently sequenced strains. Then, 1902 total genes (0% ≤ strains ≤ 100%) were annotated (Figure 1C). The core genome (99% ≤ strains ≤ 100%) comprised 1782 genes (93.69%), with 13 genes classified as soft-core genes (95% ≤ strains < 99%) (0.68%). The accessory genome (shell genes, 15% ≤ strains < 95%) and cloud genome (cloud genes, 0% ≤ strains < 15%) contained 95 (4.99%) and 12 genes (0.63%), respectively.

The phylogenetic tree revealed that *Lb. acidophilus* strains could be divided into three major clusters (Figure 1D). For strains in cluster 1, source information was mostly unknown, with no clear clustering observed based on sample region or source. Cluster 2 was enriched with strains from North America. And cluster 3 primarily contained strains originating from Asia. As for isolated source, cluster 2 was enriched with strains from food, while cluster 3 contained strains isolated from the intestine and food. Strains from this study (A2 and A3) were located within cluster 3.

### 3.2. Potential Functional Genes of the Lb. acidophilus Populations

**Figure 2:**
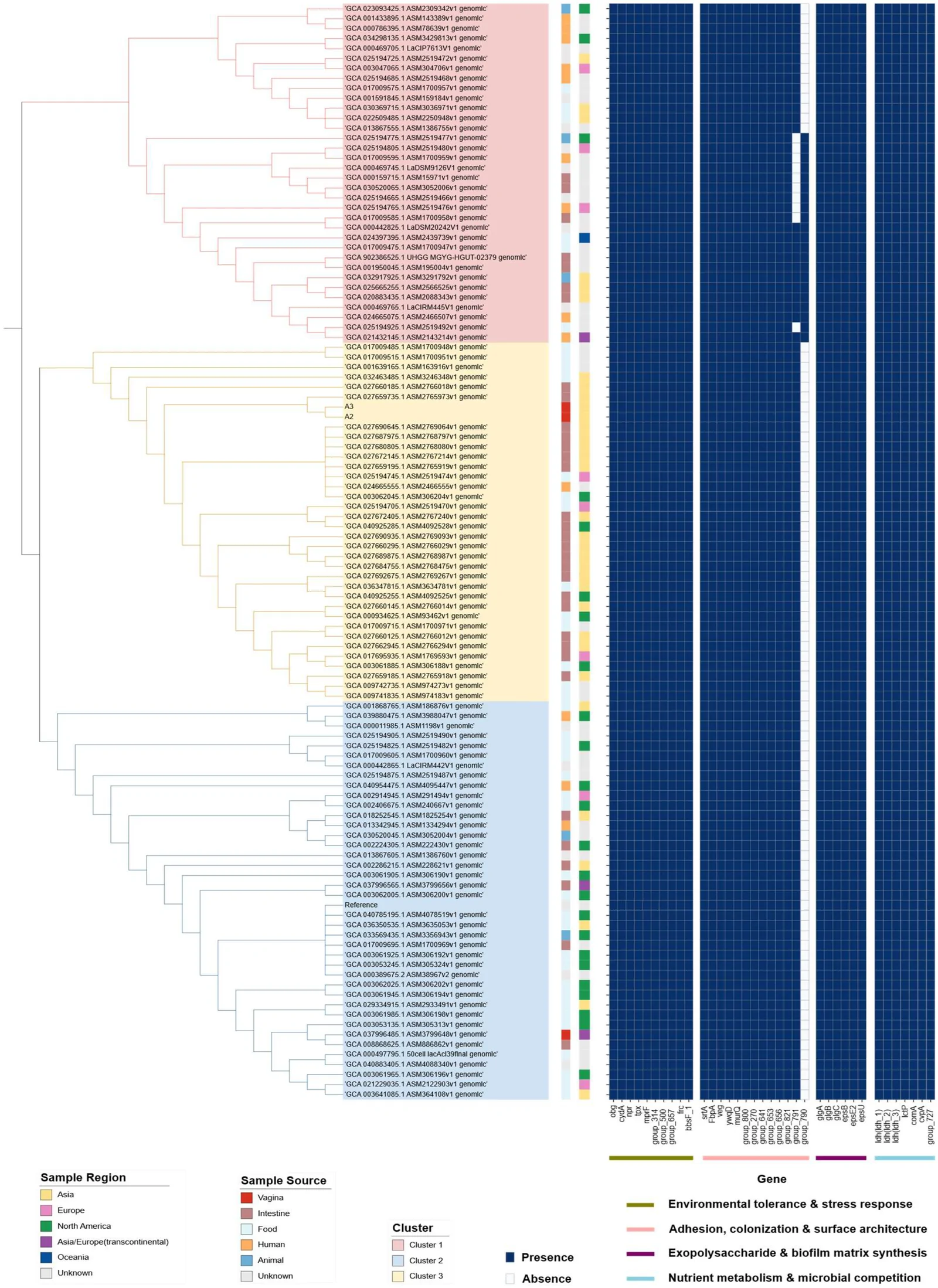
Presence–absence distribution of putative probiotic-associated gene clusters across the 109 *Lb. acidophilus* genomes. The tree on the left is the same core SNP maximum-likelihood phylogenetic tree shown in Figure 1D, displayed here in a rectangular layout.

Keyword-based screening identified 36 putative probiotic-associated gene clusters relevant to the female reproductive tract (Figure 2, Table 1). According to their annotations and potential roles in the probiotic life cycle of “survival–colonization–competitive exclusion” within the host, these clusters were assigned to four functional modules: (1) environmental tolerance and stress response; (2) adhesion, colonization, and surface architecture; (3) exopolysaccharide and biofilm matrix synthesis; and (4) nutrient metabolism and microbial competition.

Thirty-four of the 36 gene clusters were present in all 109 genomes after sequence-level verification of apparent absence calls. Of the remaining two shell gene clusters, group_791 was detected in 99 genomes (90.8%), with prevalence ranging from 80.00% to 100.00% among the known ecological origin groups. In contrast, group_790 was detected in 21 genomes (19.3%), including 0/3 vaginal genomes, 7/33 intestinal genomes, 4/13 human genomes with an unspecified anatomical source, 3/41 food-associated genomes, and 2/5 animal-associated genomes. Overall, most of the analyzed gene clusters were highly conserved, and no general ecological origin-specific distribution pattern was observed.

Notably, putative functions were represented by multiple gene clusters. Three independently annotated *ldh* clusters (*ldh_1*, *ldh_2*, and *ldh3*) were associated with lactate dehydrogenase activity (KEGG: K00016). And multiple clusters were annotated as being involved in glycogen metabolism and exopolysaccharide synthesis. Seven clusters encoding proteins with LPXTG motifs were identified, including group_270, group_641, group_653, group_790, group_791, group_821, and group_656.

Four clusters were annotated as being associated with antimicrobial activity or defense: *mprF*, *comA*, *cvpA*, and group_727. *mprF* was annotated as encoding a protein involved in cell membrane phospholipid modification, *comA* as an ABC-type bacteriocin exporter containing an N-terminal double-glycine peptidase domain, *cvpA* as a colicin V production-related protein, and group_727 as a helveticin-J-related protein. In A3, antiSMASH additionally predicted *enlA*, annotated as enterolysin A (Table S3). No known plasmid replicons were detected in A3. Its additional genomic features and predicted bacteriocin biosynthetic gene clusters are presented in Table S3 and Figure S1.

### 3.3. VF Genes and ARGs of Lb. acidophilus Population

Alignment against the VFDB database identified several genes showing sequence similarity to known virulence-associated genes, including *ppsA*/*ppsB*, *orgC*, *pefA*/*pefC*, *misL*, *hasC*, and *cap8P* (Table S4). No acquired ARGs meeting the predefined CARD screening thresholds were detected.

### 3.4. Comparative In Vitro Phenotypic Characterization of Lb. acidophilus A2 and A3

**Figure 3:**
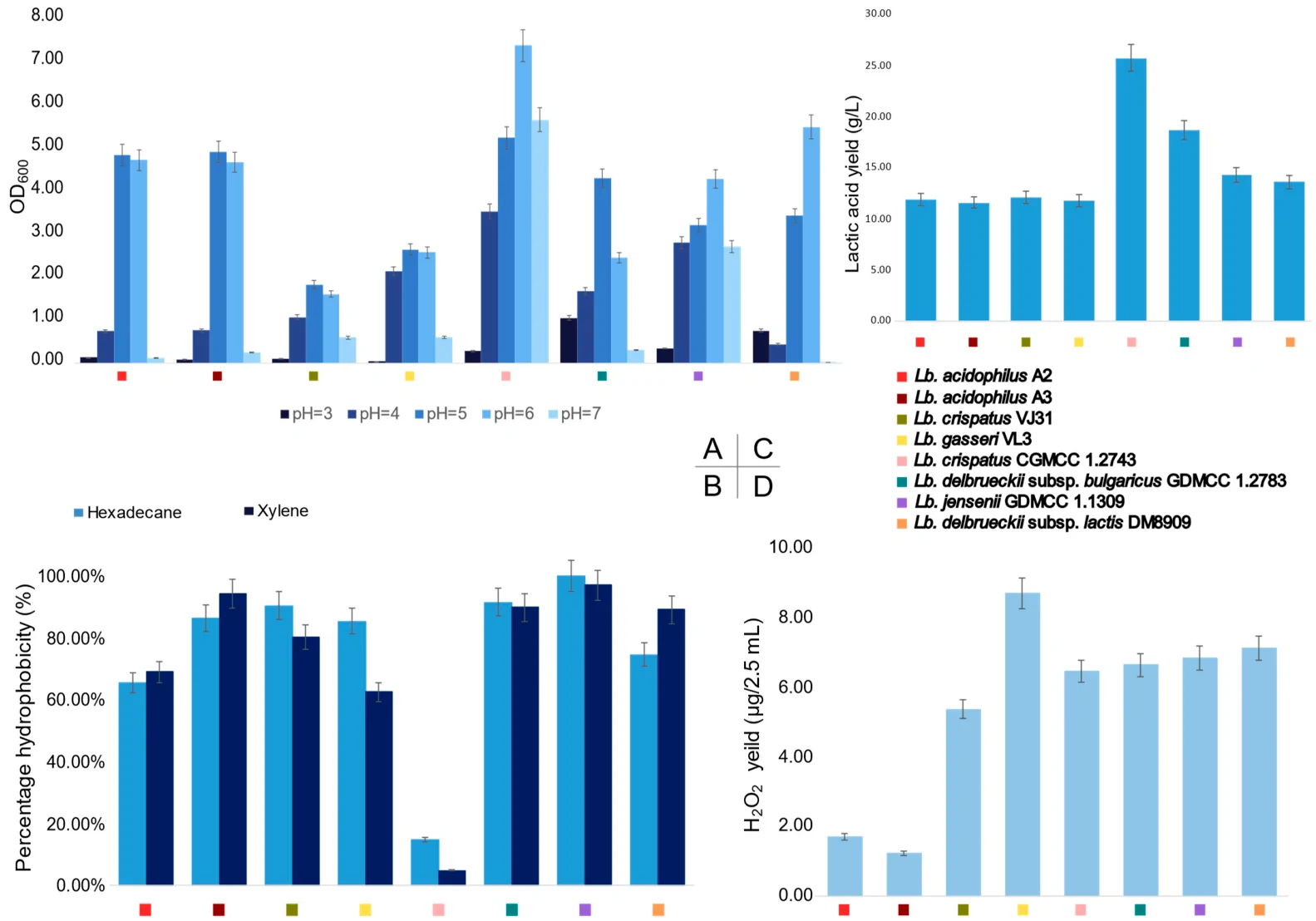
Comparative phenotypic characteristics of *Lb. acidophilus* A2 and A3 and the reference *Lactobacillus* strains. (**A**) Growth under different pH conditions. (**B**) Cell surface hydrophobicity toward hexadecane and xylene. (**C**) Lactic acid production. (**D**) H_2_O_2_ production.

The two vaginal isolates, *Lb. acidophilus* A2 and A3, were comparatively evaluated for probiotic-relevant phenotypes. Both isolates showed their highest growth at pH 5–6 and substantially weaker growth at pH 3 and 7. At pH 3, the mean OD_600_ of A2 was higher than that of A3, whereas their growth was similar at pH 4–6. At pH 7, A3 showed a higher mean OD_600_ than A2. At pH 4 (approaching the lower limit of a healthy vaginal pH) and pH 7, their growth was slightly better than that of the control strain *Lb. delbrueckii* subsp. *lactis* DM8909 (Figure 3A). The overall growth of A3 was lower than that of *Lb. crispatus* CGMCC 1.2743 and *Lb. jensenii* GDMCC 1.1309, which are dominant *Lactobacillus* species in the vagina. A3 exhibited higher cell surface hydrophobicity, expressed as bacterial adhesion to hexadecane (86.97%) and xylene (95.06%), than A2 (65.96% and 69.56%, respectively) (Figure 3B).

A2 and A3 produced comparable amounts of lactic acid, with mean values of 11.84 and 11.54 g/L, respectively. These values were similar to those observed for *Lb. crispatus* VJ31 and *Lb. gasseri* VL3 but lower than those of the higher producing reference strains. (Figure 3C). Optical density was measured at 400 nm to generate a standard curve (optical density readings per microgram of H_2_O_2_), yielding the standard curve equation y = 0.1466x − 0.025, with an R^2^ value of 0.9969, indicating that the standard curve was reliable (Figure S2). By comparing with the standard curve generated under the same conditions, both A2 and A3 produced relatively low amounts of H_2_O_2_ compared with reference strains. The calculated amount was higher for A2 (1.654 ± 0.05 μg/2.5 mL) than for A3 (1.198 ± 0.10 μg/2.5 mL) (Figure 3D).

**Figure 4:**
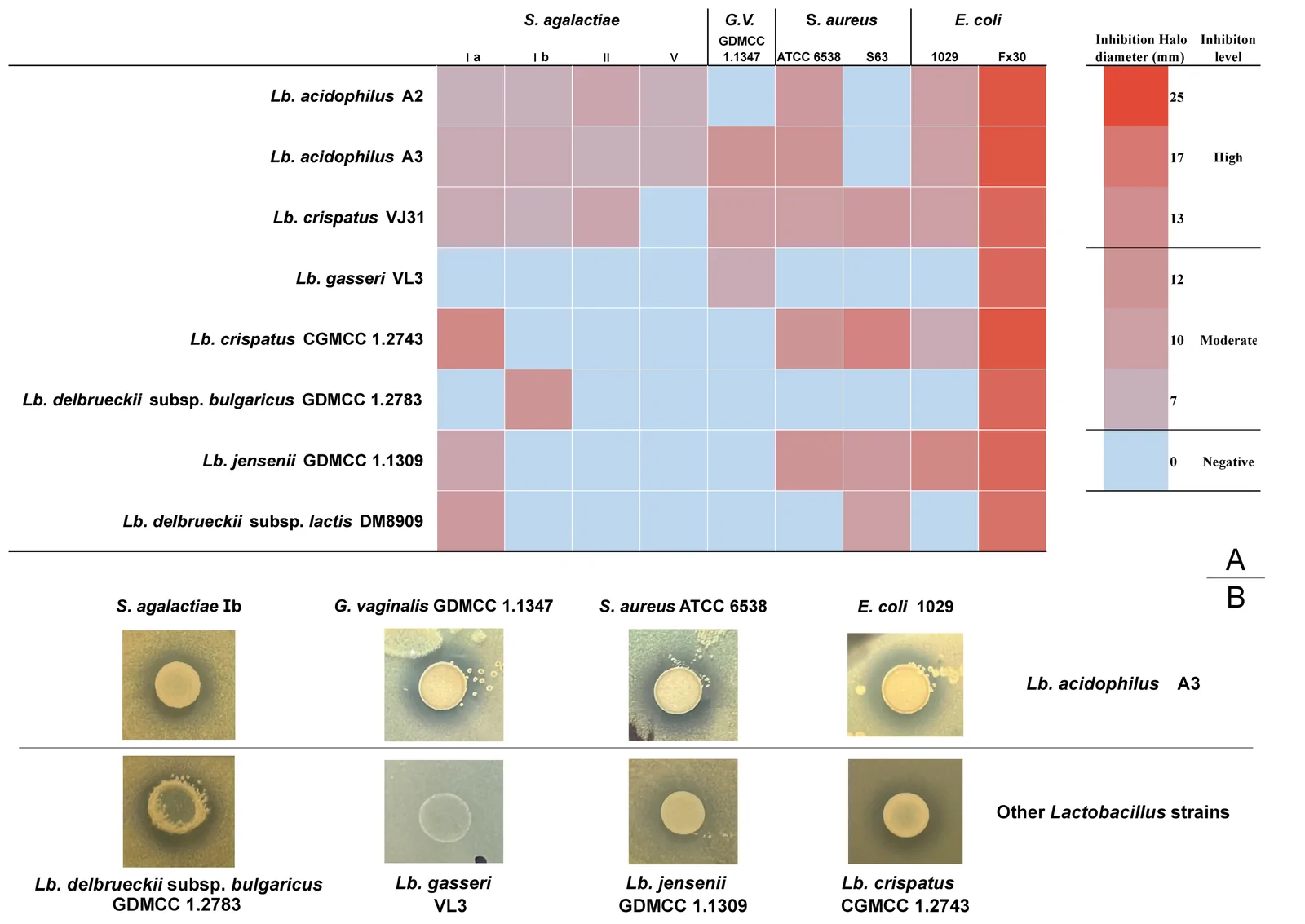
Competitive inhibition of *Lb. acidophilus* A2 and A3 and the reference *Lactobacillus* strains against reproductive tract-associated pathogens. (**A**) Heatmap showing inhibition-zone diameters against *S. agalactiae*, *G. vaginalis*, *S. aureus*, and *E. coli*. (**B**) Representative images of inhibition zones against selected indicator strains.

The antimicrobial activity of *Lb. acidophilus* A2 and A3 against female reproductive tract-associated pathogens was further assessed. A2 and A3 both exhibited high inhibitory effects against *E. coli* FX 30, with inhibition zone diameters of 23 mm, and moderate inhibition against *E. coli* 1029, *S. aureus* ATCC 6538 and all four tested *S. agalactiae* serotypes. Neither isolate inhibited *S. aureus* S63. A3 moderately inhibited *G. vaginalis* GDMCC 1.1347 (inhibition zone: 12 mm), whereas no inhibition was detected for A2 (Figure 4).

Both A2 and A3 exhibited γ-hemolysis (non-hemolytic), indicating the absence of detectable hemolytic activity. According to the applied interpretive criteria from CLSI M45, both isolates were susceptible (S) to vancomycin and linezolid and resistant (R) to clindamycin (Table 2). Both A2 and A3 exhibited daptomycin MICs of >4 μg/mL, exceeding the susceptible breakpoint defined by CLSI M45, and were therefore categorized as non-susceptible rather than resistant. MIC values for the remaining antibiotics are reported descriptively because corresponding interpretive criteria were not applied.

## 4. Discussion

This study combined population genomic analysis of 109 *Lb. acidophilus* genomes with comparative phenotypic characterization of two vaginal isolates, A2 and A3. The genomic analysis provided a population-level description of genomic diversity and the distribution of putative probiotic-associated genes, whereas the *in vitro* experiments characterized probiotic-relevant phenotypes of the two isolates.

The 109 *Lb. acidophilus* genomes had an average genome size of 1.97 Mb, which falls within the reported range for other vaginal *Lactobacillus* species [45]. Their high pairwise ANI values indicated substantial overall genomic similarity. Nevertheless, the recombination-filtered core SNP phylogeny resolved three clusters, demonstrating a detectable within-species population structure at the nucleotide level. Although some clusters contained relatively more genomes from particular geographic regions or isolation sources, genomes of different origins were distributed across the phylogeny. No clear origin-specific separation was observed. Interpretation of these patterns is also limited by incomplete metadata and unequal representation of the different source categories. Both vaginal isolates characterized in this study (A2 and A3) were located in cluster 3. The pan-genome comprised 1902 gene clusters, of which 1782 (93.69%) were classified as core genes. This core genome proportion is higher than the reported proportions for *Lactobacillus iners*, *Lb. jensenii*, *Lb. crispatus*, and *Lb. gasseri* (46.81%, 15.62%, 13.2%, and 9.83%, respectively) [45]. Although direct comparisons should be interpreted cautiously because genome sampling, quality control criteria, and pan-genome analysis methods may differ among studies. Together with the pan-genome accumulation curve, the high core proportion supports a relatively closed pan-genome and limited gene content variation within the genome collection analyzed here. This finding differs from a previous report describing an open pan-genome for *Lb. acidophilus* [46], potentially reflecting differences in dataset composition, genome quality, sample size, or analytical methodology. Therefore, the present results support a relatively conserved gene pool of *Lb. acidophilus*. However, this should not be interpreted as evidence of a particular host-adapted lifestyle or as an explanation for the abundance of *Lb. acidophilus* in the vaginal microbiota. By contrast, population genomic studies have identified open pan-genomes in *Lactiplantibacillus plantarum* and *Limosilactobacillus fermentum*, indicating a greater accumulation of accessory gene content as additional genomes are sampled [47,48].

Thirty-six gene clusters putatively associated with probiotic-relevant functions in the vaginal environment were assigned into four functional categories. Collectively, these categories encompass several processes potentially relevant to probiotic performance, including tolerance to host-associated environmental stresses [49,50], adhesion to epithelial cells or mucus [51,52], exopolysaccharide production and biofilm formation that may support bacterial persistence [53,54], and metabolic or competitive functions such as organic acid and bacteriocin production [55]. However, because these functional associations were inferred from sequence annotations, the identified clusters should be considered candidate genetic determinants rather than components of a demonstrated mechanistic pathway. Following sequence-level verification of the apparent absence calls, 34 of the 36 gene clusters were present in all 109 genomes, whereas variation was confined to the two shell gene clusters, group_790 and group_791. Their broad conservation across ecological origin groups suggests that most of the identified clusters represent widely distributed genomic features of *Lb. acidophilus*, rather than source-restricted characteristics. Group_790 was not detected among the three vaginal genomes and occurred at a relatively low frequency among food-associated genomes. Group_791 remained common across the known ecological origin groups. However, the ecological origin groups were markedly unequal in size, particularly the vaginal and animal-associated groups, and several genomes lacked detailed source information. These descriptive patterns therefore do not provide sufficient evidence of ecological adaptation. Moreover, the extensive conservation of these candidate genes indicates that gene presence or absence alone may have limited ability to predict strain-specific probiotic phenotypes. Allelic variation, gene regulation, and expression may also contribute to phenotypic differences.

Notably, multiple gene clusters annotated as members of the *ldh*-, *glg*-, and *eps*-related gene families were identified, suggesting potential functional redundancy or specialization in lactic acid production, glycogen metabolism, and exopolysaccharide biosynthesis. Such multiplicity may contribute to the robustness of these functions under changing environmental conditions, although this interpretation requires experimental validation. The conserved gene repertoire also included several putative LPXTG motif surface proteins and the acid tolerance associated gene (*bbsF_1*). These loci provide candidates for future functional investigation of host interaction and adaptation to low pH conditions.

Three bacteriocin-related gene clusters, *comA*, *cvpA*, and group_727, were conserved across all 109 *Lb. acidophilus* genomes, indicating conserved genetic potential related to bacteriocin processing, secretion, or antimicrobial protein production. This finding supports further genome-guided investigation of *Lb. acidophilus* as a potential source of antimicrobial proteins, consistent with the recent identification and experimental validation of the antibacterial protein FS25 from *Lb. acidophilus* genomic sequences [46,56]. However, *comA* and *cvpA* encode predicted bacteriocin processing, transport, or production-related proteins rather than bacteriocin structural peptides, and their genomic presence alone does not demonstrate bacteriocin production. Nevertheless, bacteriocins from different *Lb. acidophilus* strains vary considerably in molecular weight (1.1–63 kDa), thermostability (up to 50–130 °C), and pH activity range (3–10) [9,57,58,59], and their reported efficacy has been inconsistent [60,61,62,63,64,65]. Such variation may reflect differences in the producing strains and antimicrobial compounds, as well as in purification procedures, indicator organisms, and assay conditions. Moreover, probiotic activity within a complex ecosystem may result from the combined effects of bacteriocins, organic acids, H_2_O_2_, adhesion, nutrient competition, and host interactions. Thus, evaluating a single bacteriocin in isolation may not fully predict the overall probiotic effect of a strain in a complex vaginal ecosystem.

Although *Lb. acidophilus* A2 and A3 shared several probiotic-relevant properties, including lactic acid production, growth at pH 4–6, hemolysis, and antimicrobial susceptibility classifications, A3 showed higher cell surface hydrophobicity and moderate inhibition of *G. vaginalis*, whereas A2 showed no detectable inhibition. Similar strain-level variation has been reported among vaginal lactobacilli. Differences have been observed among vaginal isolates on the basis of multiple phenotypic criteria [48,66]. Therefore, the present comparison provides a practical basis for prioritizing A3 for further evaluation, particularly for vaginal application. However, cell surface hydrophobicity is only an indirect indicator of adhesion, and the inhibition of *G. vaginalis* should be confirmed in replicated assays. The results also do not identify a single mechanism responsible for the observed antimicrobial activity. The higher H_2_O_2_ production of *Lb. gasseri* VL3 was not accompanied by broader antimicrobial activity. This is consistent with reports that the antimicrobial activity of H_2_O_2_ is strongly dependent on the experimental and vaginal conditions [67]. It may also depend on the target organism and assay conditions. Similarly, bacteriocin-mediated activity also remains plausible. Bacteriocin-containing extract from *Lb. acidophilus* strain inhibited *G. vaginalis* and *S. agalactiae* after organic acids and H_2_O_2_ were neutralized [60]. However, the bacteriocin-related loci detected in A2 and A3, together with the additional prediction of *enlA* in A3, do not demonstrate gene expression or establish causality. The inhibition observed here may therefore result from the combined effects of acidification, H_2_O_2_, bacteriocins, and other secreted metabolites. Direct epithelial adhesion assays and targeted analysis of antimicrobial metabolites will be required to determine whether the apparent advantages of A3 are reproducible and mechanistically relevant.

Although reproductive tract *Lactobacillus* strains may possess acid tolerance determinants [68] and *Lb. acidophilus* is known to show robust *in vitro* viability [9], both A2 and A3 showed only limited growth at pH 3.0. This finding indicates that acid tolerance potential does not necessarily confer robust proliferation under severe acid stress. Although inhibition by self-produced acidic metabolites has previously been reported [54], the endpoint growth assay used here cannot distinguish this effect from the direct influence of the initially low environmental pH. Thus, the results indicate limited proliferation under severe acid stress but do not demonstrate a complete lack of acid tolerance.

No acquired ARGs meeting the predefined CARD thresholds were detected among the 109 *Lb. acidophilus* genomes, reducing concern regarding known transferable resistance determinants. Intrinsic resistance to aminoglycosides and quinolones is well documented in many *Lactobacillus* species [69,70,71]. However, *Lb. acidophilus* is generally susceptible to vancomycin [72,73,74], consistent with the susceptibility of A2 and A3 to vancomycin and linezolid. Both isolates showed resistance to clindamycin, which has also been reported [73,75]. Both were non-susceptible to daptomycin. For antimicrobial agents without applicable interpretive criteria, the MIC values were considered descriptive rather than classified as susceptible or resistant. The absence of detected acquired ARGs is relevant given previous reports of multidrug resistance and transferable resistance determinants in commercial probiotic isolates [69,76,77], although it does not alone establish probiotic safety. Although VFDB screening identified several sequences homologous to virulence-associated genes, manual examination indicated that these sequences mainly encoded proteins involved in primary metabolism or cell surface architecture. Because sequence homology alone does not demonstrate pathogenic function, these matches were not considered evidence of classical functional virulence determinants. This is not uncommon in lactobacilli [78]. Together with the non-hemolytic phenotypes of A2 and A3, these findings provide preliminary genomic and phenotypic support for their safety, while the clindamycin and daptomycin results warrant cautious interpretation.

Limitations of this study include the reliance on genomic predictions and *in vitro* assays, the unequal sizes and incomplete metadata of the ecological origin groups, and the phenotypic characterization of only two vaginal isolates. The expression and activity of the predicted genes, interactions within polymicrobial communities, *in vivo* colonization, safety, protective efficacy, long-term stability, and optimal dosage and administration route remain to be addressed. These aspects should be investigated before clinical application is considered.

## 5. Conclusions

This study characterized the pan-genome and distribution of putative probiotic-associated genes across 109 *Lb. acidophilus* genomes, and comparatively evaluated probiotic-relevant phenotypes in two vaginal isolates, A2 and A3. The population exhibited a highly conserved closed pan-genome, and 34 of the 36 putative probiotic-associated gene clusters were present in all 109 genomes. Despite broadly similar phenotypes, A3 showed higher cell surface hydrophobicity than A2 and inhibited *G. vaginalis*, illustrating strain-dependent variation within a conserved genomic background. These features support its prioritization for further preclinical evaluation. However, replicated phenotypic assays, mechanistic studies, and *in vivo* validation are required before any clinical development of A3 as a biotherapeutic candidate for preventing or treating BV.

## Figures and Tables

**Table 1:** Functional genes of *Lb. acidophilus*.

Main Category	Sub-Category	Annotation	Description
Environmental tolerance and stress response	Cell Cycle and Stress Response	*obg*	An essential GTPase that binds GTP, GDP and possibly (p)ppGpp with moderate affinity, with high nucleotide exchange rates and a fairly low GTP hydrolysis rate. Plays a role in control of the cell cycle, stress response, ribosome biogenesis and in those bacteria that undergo differentiation, in morphogenesis control
	pH Adaptation and Acid Tolerance	*bbsF_1*	Involved in the catabolism of oxalate and in the adaptation to low pH via the induction of the oxalate-dependent acid tolerance response (ATR). Catalyzes the transfer of the CoA moiety from formyl-CoA to oxalate
		*frc*	Involved in the catabolism of oxalate and in the adaptation to low pH via the induction of the oxalate-dependent acid tolerance response (ATR). Catalyzes the transfer of the CoA moiety from formyl-CoA to oxalate
	Oxidative Stress Response	*cydA*	ubiquinol oxidase
		*npr*	NADH oxidase
		*tpx*	Thiol-specific peroxidase that catalyzes the reduction of hydrogen peroxide and organic hydroperoxides to water and alcohols, respectively. Plays a role in cell protection against oxidative stress by detoxifying peroxides
	Cofactor Metabolism	group_314, group_500, group_657	Pyridoxamine 5′-phosphate oxidase
	Membrane Modification and Antibiotic Tolerance	*mprF*	Catalyzes the transfer of a lysyl group from L-lysyl- tRNA (Lys) to membrane-bound phosphatidylglycerol (PG), which produces lysyl-phosphatidylglycerol (LPG), a major component of the bacterial membrane with a positive net charge. LPG synthesis contributes to bacterial virulence as it is involved in the resistance mechanism against cationic antimicrobial peptides (CAMPs) produced by the host’s immune system (defensins, cathelicidins) and by the competing microorganisms
Adhesion, colonization and surface architecture	Fibronectin Binding	*FbpA*	Fibronectin-binding protein
	Surface Protein Anchoring	*srtA*	Sortase family
		group_270, group_641, group_653, group_790, group_791, group_821	LPXTG motif cell wall anchor domain protein
		group_656	LPXTG cell wall anchor motif
	Adhesins and Colonization Factors	group_800	Putative adhesin
		*veg*	Biofilm formation stimulator VEG
	Capsule and Surface Polysaccharides	*ywqD*	Capsular exopolysaccharide family
	Cell Wall Synthesis and Modification	*murQ*	Specifically catalyzes the cleavage of the D-lactyl ether substituent of MurNAc 6-phosphate, producing GlcNAc 6-phosphate and D-lactate
Exopolysaccharide and biofilm matrix synthesis	Glycogen Synthesis and Metabolism	*glgA*	Synthesizes alpha-1,4-glucan chains using ADP-glucose
		*glgB*	Catalyzes the formation of the alpha-1,6-glucosidic linkages in glycogen by scission of a 1,4-alpha-linked oligosaccharide from growing alpha-1,4-glucan chains and the subsequent attachment of the oligosaccharide to the alpha-1,6 position
		*glgC*	Catalyzes the synthesis of ADP-glucose, a sugar donor used in elongation reactions on alpha-glucans
	Exopolysaccharide Synthesis Machinery	*epsB*	biosynthesis protein
		*epsE2*	Bacterial sugar transferase
		*epsU*	Polysaccharide biosynthesis protein
Nutrient metabolism and microbial competition	Lactate Metabolism	*ldh (ldh_1)*	Belongs to the LDH MDH superfamily
		*ldh (ldh_2)*	Belongs to the LDH MDH superfamily
		*ldh3*	Lactate/malate dehydrogenase, alpha/beta C-terminal domain
		*lctP*	L-lactate permease
	Bacteriocin Synthesis and Secretion	*comA*	ABC-type bacteriocin lantibiotic exporters, contain an N-terminal double-glycine peptidase domain
		*cvpA*	Colicin V production protein
		group_727	Bacteriocin helveticin-J

**Table 2:** MICs and antimicrobial susceptibility interpretations of *Lb. acidophilus* A2 and A3.

Antibiotics	Abbreviation	A2 MIC (μg/mL)	A2 Interpretation	A3 MIC (μg/mL)	A3 Interpretation
Azithromycin	AZI	2	—	2	—
Amikacin	AMI	>32	—	>32	—
Trimethoprim sulfamethoxazole	SXT	8/152	—	8/152	—
Vancomycin	VAN	1	S ^1^	1	S ^1^
Rifampin	RIF	1	—	0.5	—
Gentamicin	GEN	32	—	32	—
Linezolid	LZD	4	S	2	S
Clindamycin	CLI	4	R ^2^	4	R ^2^
Oxacillin + 2%NaCl	OXA+	0.5	—	0.5	—
Nitrofurantoin	NIT	>128	—	>128	—
Daptomycin	DAP	>4	NS ^3^	>4	NS ^3^
Florfenicol	FFN	<1	—	4	—

^1^ S, susceptible; ^2^ R, resistant; ^3^ NS, non-susceptible; —, no interpretive criterion available for the corresponding antimicrobial organism combination in CLSI M45.

## Data Availability

Sequence data are publicly available at NCBI. Both the genome assemblies and the raw sequencing reads (FASTQ) for strain *Lb. acidophilus* A3 and A2 have been deposited in the NCBI database under BioProject PRJNA1474769, with BioSample Accession and accession number of SAMN60609292, SAMN60609291 and GCA_058148475.1, GCA_058147065.1, respectively.

## Supplementary Materials

The following supporting information can be downloaded at: https://www.mdpi.com/article/10.3390/genes17091009/s1, Table S1: Experimental strains information; Table S2: Genome accession and origin metadata for 109 *Lb. acidophilus* strains; Table S3: Genomic information of *Lb. acidophilus* A3; Table S4: Virulence gene annotation results against the VFDB database (identity ≥ 80%); Figure S1: Genomic features of *Lb. acidophilus* A3; Figure S2: Standard curve of H_2_O_2_ production.

## Abbreviations

The following abbreviations are used in this manuscript:

RTIs	Reproductive tract infections
BV	Bacterial vaginosis
G.V.	*Gardnerella vaginalis*
CGMCC	China General Microbiological Culture Collection Center
GDMCC	Guangdong Microbial Culture Collection Center
ATCC	American Type Culture Collection
CDS	Coding sequences
ANI	Average nucleotide identity
H_2_O_2_	Hydrogen peroxide
PPB	Potassium phosphate buffer
CFU	Colony-forming unit
VF	Virulence factor
ARGs	Antibiotic resistance genes
MIC	Minimum inhibitory concentration
MRS	de Man, Rogosa and Sharpe
