# Supplementary material for: Vaginal Probiotic Potential of Lactobacillus acidophilus: Population Genomic and Phenotypic Analysis
Source: Genes (Basel). 2026 Aug 26;17(9):1009. doi: 10.3390/genes17091009 (PMC13606125; doi:10.3390/genes17091009)
Supplement: Supplementary file 1 [file genes-17-01009-s001.zip › genes-4460250-supplementary.pdf]

Supplementary Material

Vaginal probiotic potential of *Lactobacillus acidophilus*: population genomic and phenotypic analysis

Yixin Mao[1, 2, 3], Yanqing Che[1, 4], Mengjie Li[1, 4], Guodong Yan[1, 5], Xiao Liu[1], Ruocheng Yang[1, 5], Hongzhou Li[1, 4], Yeshun Fan[1], Haojie Zhan[1, 4], Zhiwen Sun[1], Xuemei Bai[1], He Gao[1] and Duochun Wang[1,*]

1. *National Key Laboratory of Intelligent Tracking anfd Forecasting for Infectious Diseases, National Institute for Communicable Disease Control and Prevention, Chinese Center for Disease Control and Prevention & Chinese Academy of Preventive Medicine, Beijing 102206, China*
2. *National Key Laboratory of Intelligent Tracking and Forecasting for Infectious Diseases, National Institute of Environmental Health, Chinese Center for Disease Control and Prevention & Chinese Academy of Preventive Medicine, Beijing 100021, China*
3. *China CDC Key Laboratory of Environment and Population Health, National Institute of Environmental Health, Chinese Center for Disease Control and Prevention & Chinese Academy of Preventive Medicine, Beijing 100021, China*
4. *School of Public Health, Lanzhou University, Lanzhou, Gansu 730000, China*
5. *School of Public Health, Gansu University of Chinese Medicine, Lanzhou, Gansu 730000, China*

*Corresponding author:

Duochun Wang. Changbai Road 155, Changping, Beijing 102206, China. E-mail address: wangduochun@icdc.cn

Table of Contents

Table S1 Experimental strains information

Table S2 Genome accession and origin metadata for 109 *Lb. acidophilus* strains

Table S3 Genomic information of *Lb. acidophilus A3*

Table S4 Virulence gene annotation results against the VFDB database (identity ≥ 80%)

Figure S1 Genomic features of Lb. *acidophilus A3*

Figure S2 Standard curve of H2O2 production

Table S1 Experimental strains information

Strain ID	Species	Type	Source	Isolation source	Purpose
A2	*Lactobacillus acidophilus*	Common	China CDC	Female reproductive tract	Study isolate
A3	*Lactobacillus acidophilus*	Common	China CDC	Female reproductive tract	Study isolate
VJ31	*Lactobacillus crispatus*	Common	China CDC	Female reproductive tract	Reference
VL3	*Lactobacillus gasseri*	Common	China CDC	Female reproductive tract	Reference
CGMCC 1.2743	*Lactobacillus crispatus*	Common	CGMCC	Healthy vagina	Reference
GDMCC 1.2783	*Lactobacillus delbrueckii* subsp. *bulgaricus*	Common	GDMCC	NA	Reference
GDMCC 1.1309	*Lactobacillus jensenii*	Common	GDMCC	Human vaginal secretions	Reference
DM8909	*Lactobacillus delbrueckii* subsp. *lactis*	Common	Medicine	Vaginal tract of healthy adult woman	Reference
Ia	*Streptococcus agalactiae*	Serotype Ia	China CDC	Clinical isolate	Indicator
Ib	*Streptococcus agalactiae*	Serotype Ib	China CDC	Clinical isolate	Indicator
II	*Streptococcus agalactiae*	Serotype II	China CDC	Clinical isolate	Indicator
V	*Streptococcus agalactiae*	Serotype V	China CDC	Clinical isolate	Indicator
GDMCC 1.1347	*Gardnerella vaginalis*	Common	GDMCC	Human vaginal secretions	Indicator

Strain ID	Species	Type	Source	Isolation source	Purpose
ATCC 6538	*Staphylococcus aureus*	Common	China CDC	Lesion	Indicator
S63	*Staphylococcus aureus*	Enterotoxigenic	China CDC	Clinical isolate	Indicator
1029	*Escherichia coli*	Common	China CDC	Clinical isolate	Indicator
Fx30	*Escherichia coli*	O104:H4	China CDC	Clinical isolate	Indicator
ATCC 25922	*Escherichia coli*	Common	China CDC	Clinical isolate	AST Control

Note: CGMCC, China General Microbiological Culture Collection Center; GDMCC, Guangdong Microbial Culture Collection Center; ATCC, American Type Culture Collection; NA, not provided.

Table S2 Genome accession and origin metadata for 109 *Lb. acidophilus* strains

Accession number	Genome size (Mb)	GC (%)	Collection date	Geographic origin	Isolation source
GCA_058147065.1 (A2)	1.9668	34.57	2023/9	China: Beijing	vaginal specimen
GCA_058148475.1 (A3)	1.9675	34.57	2023/9	China: Beijing	vaginal specimen
GCA_000011985.1	1.9936	34.5	Unknown	Unknown	Unknown
GCA_000159715.1	2.0205	34.5	Unknown	Unknown	human gastrointestinal tract
GCA_000389675.2	1.9916	34.5	Unknown	Unknown	Unknown
GCA_000442825.1	2.0479	34.5	Unknown	Unknown	Unknown
GCA_000442865.1	1.9870	34.5	Unknown	Unknown	Unknown
GCA_000469705.1	1.9518	34.5	Unknown	Unknown	Unknown
GCA_000469745.1	1.9918	34.5	Unknown	Unknown	Unknown
GCA_000469765.1	2.0020	34.5	Unknown	Unknown	Unknown
GCA_000497795.1	1.9710	34.5	2012	Unknown	commercially purchased yogurt
GCA_000786395.1	1.9567	34.5	1931	Unknown	human
GCA_000934625.1	1.9920	34.5	2002	USA: Maryland	yogurt
GCA_001433895.1	1.9540	34.5	1970	Unknown	human
GCA_001591845.1	1.9553	34.5	Unknown	Unknown	Unknown
GCA_001639165.1	1.9517	34.5	Unknown	Unknown	yogurt fermentation
GCA_001868765.1	1.9923	34.5	2003/9/1	China: Sinkiang	homemade cheese
GCA_001950045.1	2.0095	34.5	Unknown	Unknown	healthy infant feces
GCA_002224305.1	1.9919	34.5	1986	USA	infant rectal swab(feces?)
GCA_002286215.1	1.9912	34.5	2015/8/25	South Korea: Seoul	human feces

Accession number	Genome size (Mb)	GC (%)	Collection date	Geographic origin	Isolation source
GCA_002406675.1	2.0468	35.5	2016/11/7	Canada: Montreal, Quebec	commercial probiotic
GCA_002914945.1	1.9657	34.5	2017	Italy	food product - supplement
GCA_003047065.1	2.0100	34.5	1970	France	human
GCA_003053135.1	1.9684	34.5	2016/11/5	USA:MD	commercial dietary supplements
GCA_003053245.1	1.9707	34.5	2016/1/10	USA:MD	commercial dietary supplements
GCA_003061885.1	1.9695	34.5	2016/7/27	USA:MD	commercial dietary supplements
GCA_003061905.1	1.9639	34.5	2016/5/7	USA:MD	commercial dietary supplements
GCA_003061925.1	1.9690	34.5	2016/1/23	USA:MD	commercial dietary supplements
GCA_003061945.1	1.9676	34.5	2016/1/10	USA:MD	commercial dietary supplements
GCA_003061965.1	1.9643	34.5	2016/5/7	USA:MD	commercial dietary supplements
GCA_003061985.1	1.9688	34.5	2016/2/24	USA:MD	commercial dietary supplements
GCA_003062005.1	1.9820	34.5	2016/3/12	USA:MD	commercial dietary supplements
GCA_003062025.1	1.9685	34.5	2015/12/12	USA:MD	commercial dietary supplements
GCA_003062045.1	1.9701	34.5	2016/1/13	USA:MD	commercial dietary supplements
GCA_003641085.1	1.9510	34.5	2008/9/26	India: Hyderabad	fermented food
GCA_008868625.1	1.9698	34.5	Unknown	Unknown	adult feces
GCA_009741835.1	1.9626	34.5	Unknown	Unknown	dairy product
GCA_009742735.1	1.9627	34.5	Unknown	Unknown	dairy product
GCA_013342945.1	1.9920	34.5	Unknown	Unknown	human
GCA_013867555.1	1.9533	34.5	2020/7/20	Unknown	Unknown
GCA_013867605.1	1.9658	34.5	2020/7/20	Unknown	Unknown
GCA_017009475.1	1.9878	34.5	Unknown	Unknown	commercial yoghurt
GCA_017009485.1	1.9697	34.5	Unknown	Unknown	cheese starter
GCA_017009515.1	1.9673	34.5	Unknown	Unknown	commercial culture

Accession number	Genome size (Mb)	GC (%)	Collection date	Geographic origin	Isolation source
GCA_017009575.1	1.9501	34.5	Unknown	Unknown	acidophilus milk
GCA_017009585.1	2.0036	34.5	Unknown	Unknown	human intestine
GCA_017009595.1	1.9777	34.5	Unknown	Unknown	human
GCA_017009605.1	1.9666	34.5	Unknown	Unknown	sweet acidophilus milk
GCA_017009695.1	1.9618	34.5	Unknown	Unknown	infant feces
GCA_017009715.1	1.9654	34.5	Unknown	Unknown	commercial product
GCA_017695935.1	1.9767	34.5	2014/2/22	Ireland: Cork	infant feces
GCA_018252545.1	1.9634	34.5	2009	China: Bama	feces
GCA_020883435.1	2.0374	35	2021/8/1	China: Jinan	human gut
GCA_021229035.1	1.9639	34.5	1989	Bulgaria: Haskov	sample from fruit (melon)
GCA_021432145.1	2.0309	34.5	2021	Russia	human
GCA_022509485.1	1.9533	34.5	2018/1/1;2021/1/1	Pakistan: Islamabad	fermented milk product (Dahi)
GCA_023093425.1	1.9598	34.5	2021/2/5	Canada: London	cow manure (cattle)
GCA_024397395.1	2.0406	34.5	Unknown	New Zealand	yoghurt
GCA_024665075.1	2.0310	34.5	Unknown	Unknown	human
GCA_024665555.1	1.9920	34.5	Unknown	Unknown	human
GCA_025194665.1	1.9997	34.5	1969	Unknown	Unknown
GCA_025194685.1	1.9543	34.5	1990	Unknown	human
GCA_025194705.1	1.9662	34.5	2001	France	fermented milk
GCA_025194725.1	1.9542	34.5	2000	Mongolia	Unknown
GCA_025194745.1	1.9660	34.5	1996	France	fermented milk
GCA_025194765.1	1.9895	34.5	1996	France	human
GCA_025194775.1	1.9843	34.5	1992	USA	rat intestine
GCA_025194805.1	1.9959	34.5	1996	United Kingdom	Unknown

Accession number	Genome size (Mb)	GC (%)	Collection date	Geographic origin	Isolation source
GCA_025194825.1	1.9511	34.5	1996	USA	fermented milk
GCA_025194875.1	1.9665	34.5	1990	Unknown	fermented milk
GCA_025194905.1	1.9521	34.5	1990	Unknown	fermented milk
GCA_025194925.1	2.0031	34.5	1990	Unknown	fermented milk
GCA_025665255.1	2.0311	34.5	2020/8/1	China: Shandong	human gut
GCA_027659185.1	1.9638	34.5	2014/2/1	China: Shenzhen	human fecal material
GCA_027659195.1	1.9629	34.5	2014/2/1	China: Shenzhen	human fecal material
GCA_027659735.1	1.9561	34.5	2014/2/1	China: Shenzhen	human fecal material
GCA_027660125.1	1.9557	34.5	2014/2/1	China: Shenzhen	human fecal material
GCA_027660145.1	1.9582	34.5	2014/2/1	China: Shenzhen	human fecal material
GCA_027660185.1	1.9556	34.5	2014/2/1	China: Shenzhen	human fecal material
GCA_027660295.1	1.9565	34.5	2014/2/1	China: Shenzhen	human fecal material
GCA_027662945.1	1.9707	34.5	2014/2/1	China: Shenzhen	human fecal material
GCA_027672145.1	1.9659	34.5	2014/2/1	China: Shenzhen	human fecal material
GCA_027672405.1	1.9642	34.5	2014/2/1	China: Shenzhen	human fecal material
GCA_027680805.1	1.9584	34.5	2014/2/1	China: Shenzhen	human fecal material
GCA_027684755.1	1.9522	34.5	2014/2/1	China: Shenzhen	human fecal material
GCA_027687975.1	1.9586	34.5	2014/2/1	China: Shenzhen	human fecal material
GCA_027689875.1	1.9558	34.5	2014/2/1	China: Shenzhen	human fecal material
GCA_027690645.1	1.9533	34.5	2014/2/1	China: Shenzhen	human fecal material
GCA_027690935.1	1.9554	34.5	2014/2/1	China: Shenzhen	human fecal material
GCA_027692675.1	1.9589	34.5	2014/2/1	China: Shenzhen	human fecal material
GCA_029334915.1	1.9672	34.5	2021/10/12	Bangladesh	yogurt
GCA_030369715.1	1.9789	34.5	2011/1/1	China	fermented yoghurt

Accession number	Genome size (Mb)	GC (%)	Collection date	Geographic origin	Isolation source
GCA_030520045.1	1.9920	34.5	Unknown	Unknown	rectum of pig
GCA_030520065.1	2.0355	35	Unknown	Unknown	human gastrointestinal tract
GCA_032463485.1	1.9920	34.5	2021/9/16	China: Ningbo	fermented milk
GCA_032917925.1	2.0309	34.5	2022/7/28	China: Inner Mongolia Autonomous Region	Weathered cattle and sheep bones
GCA_033569435.1	1.9916	34.5	2014/10/1	USA: North Carolina	gallus
GCA_034298135.1	1.9788	34.5	2017/7/17	USA	human
GCA_036347815.1	1.9569	34.5	2013/4/1	India: Maharashtra	dairy product
GCA_036350535.1	1.9630	34.5	2013/4/1	India: Maharashtra	dairy product
GCA_037996485.1	2.0023	35	2001/3/6	Turkey: Izmir, Cigli	vaginal specimen
GCA_037996565.1	2.0000	35	2001/2/1	Turkey: Istanbul, Sisli	human intestinal rectoscopic specimen
GCA_039880475.1	1.9920	34.5	2020/2/2	USA	human
GCA_040785195.1	1.9684	34.5	2021	USA: Glendale, Arizona	probiotic pill
GCA_040883405.1	1.9472	34.5	Unknown	Unknown	Unknown
GCA_040925255.1	1.9494	34.5	2021/4/1	USA: New York	human feces
GCA_040925285.1	1.9641	34.5	2021/4/1	USA: New York	human feces
GCA_040954475.1	1.9920	34.5	2021	USA	human
GCA_902386525.1	2.0095	34.5	Unknown	Unknown	human gut

Table S3 Genomic information of *Lb. acidophilus* A3

Genome size (Mb)	GC content (%)	CDS	rRNA	tRNA	Prophage	Plasmid	Insertion sequence (IS)	Putative probiotic-associated gene clusters identified	Predicted bacteriocin-related genes	Predicted ARO Term (with % identity of matching region, and % length of reference sequence)
1.97	35.1	1849	4	61	0	0	14	35/36	*comA, cvpA, hlvJ*, *enlA*	*vanT* gene in *vanG* cluster (30.29, 52.81)

Table S4 Virulence gene annotation results against the VFDB database (identity ≥ 80%)

Sequence name	Target ID	Identity (%)	Alignment length; Mismatches; Gaps	E-value	Bit score	Gene
GCA_017695935.1_ASM1769593v1_genomic	VFG007823(gb|NP_217447)	100	80; 0; 0	6.62e-45	150	*ppsA*
GCA_017695935.1_ASM1769593v1_genomic	VFG007832(gb|NP_217448)	100	55; 0; 0	1.0500000000000001e-32	114	*ppsB*
GCA_002406675.1_ASM240667v1_genomic	VFG003635(gb|NP_461789)	98.4	129; 2; 0	3.06e-88	250	*orgC*
GCA_002406675.1_ASM240667v1_genomic	VFG000434(gb|NP_490509)	98	50; 1; 0	6.59e-26	94.7	*pefC*
GCA_027692675.1_ASM2769267v1_genomic	VFG000964(gb|WP_010922799)	97.7	43; 1; 0	2.67e-22	83.2	*hasC*
GCA_027660295.1_ASM2766029v1_genomic	VFG000964(gb|WP_010922799)	97.6	41; 1; 0	4.850000000000001e-21	79.7	*hasC*
GCA_002406675.1_ASM240667v1_genomic	VFG002304(gb|NP_462656)	97.4	117; 3; 0	2.67e-74	234	*misL*
GCA_003061905.1_ASM306190v1_genomic	VFG001312(gb|WP_000723452)	84.2	19; 3; 0	1.85e-06	38.1	*cap8P*
GCA_003061965.1_ASM306196v1_genomic	VFG001312(gb|WP_000723452)	84.2	19; 3; 0	1.85e-06	38.1	*cap8P*
GCA_025194665.1_ASM2519466v1_genomic	VFG001312(gb|WP_000723452)	84.2	19; 3; 0	2.49e-06	38.1	*cap8P*
GCA_025194685.1_ASM2519468v1_genomic	VFG001312(gb|WP_000723452)	84.2	19; 3; 0	2.49e-06	38.1	*cap8P*
GCA_025194705.1_ASM2519470v1_genomic	VFG001312(gb|WP_000723452)	84.2	19; 3; 0	2.49e-06	38.1	*cap8P*
GCA_025194725.1_ASM2519472v1_genomic	VFG001312(gb|WP_000723452)	84.2	19; 3; 0	2.49e-06	38.1	*cap8P*
GCA_025194745.1_ASM2519474v1_genomic	VFG001312(gb|WP_000723452)	84.2	19; 3; 0	2.49e-06	38.1	*cap8P*
GCA_025194775.1_ASM2519477v1_genomic	VFG001312(gb|WP_000723452)	84.2	19; 3; 0	2.49e-06	38.1	*cap8P*
GCA_025194825.1_ASM2519482v1_genomic	VFG001312(gb|WP_000723452)	84.2	19; 3; 0	2.49e-06	38.1	*cap8P*
GCA_025194875.1_ASM2519487v1_genomic	VFG001312(gb|WP_000723452)	84.2	19; 3; 0	2.49e-06	38.1	*cap8P*
GCA_025194925.1_ASM2519492v1_genomic	VFG001312(gb|WP_000723452)	84.2	19; 3; 0	2.49e-06	38.1	*cap8P*
GCA_036347815.1_ASM3634781v1_genomic	VFG001312(gb|WP_000723452)	84.2	19; 3; 0	1.85e-06	38.1	*cap8P*
GCA_036350535.1_ASM3635053v1_genomic	VFG001312(gb|WP_000723452)	84.2	19; 3; 0	1.85e-06	38.1	*cap8P*
GCA_002406675.1_ASM240667v1_genomic	VFG000435(gb|NP_490510)	80.6	165; 30; 2	5.879999999999999e-82	237	*pefA*

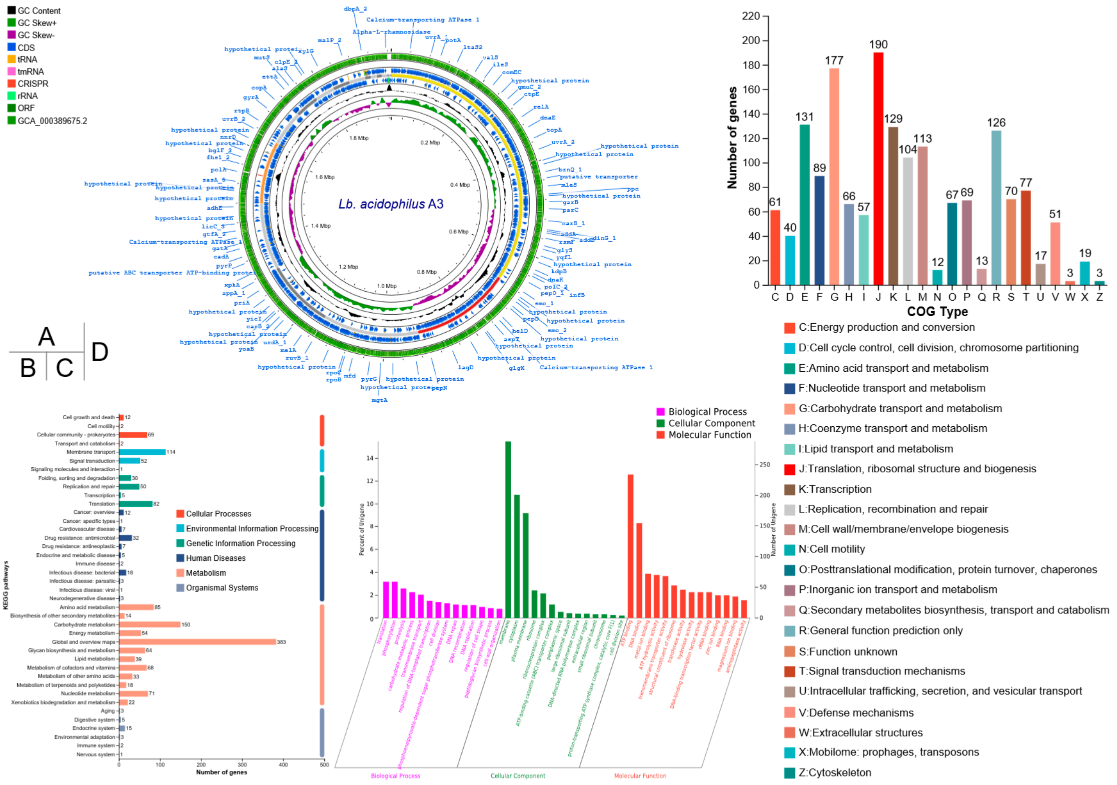

Figure S1 Genomic features of *Lb. acidophilus* A3

(A) Whole-genome map of *Lb. acidophilus* A3 with coding sequences (CDS) compared to the reference genome (GCA_000389675.2) (B) KEGG pathway annotation (C) GO pathway annotation (D) COG functional classification.

The genome size of *Lb. acidophilus* A3 is 1.97 Mb (1,967,478 bp), with a G+C content (GC content in gene regions) of 35.1% (Figure S1A). The genome contains 14 insertion sequences, 17 tandem repeat sequences, 61 tRNAs, and 4 rRNAs (including one 16S rRNA, one 23S rRNA, and two 5S rRNAs), as well as 41 sRNAs. No known plasmid replicon detected. Functional annotation and enrichment analysis of the whole genome of *Lb. acidophilus* A3 revealed that its gene functions are primarily enriched in categories such as global and overview maps, carbohydrate metabolism, carbohydrate transport and metabolism, amino acid transport and metabolism, membrane transport, transcription, ATP binding, DNA binding, translation, general function prediction, and ribosomal structure and biogenesis (Figure S1B, C, D). Among the 36 putative probiotic-associated gene clusters identified at the population level, 35 were detected in A3 (Table S3). COG functional classification of the whole A3 genome showed that genes were primarily enriched in categories S (Function unknown), M (Cell wall/membrane/envelope biogenesis), and C (Energy production and conversion).

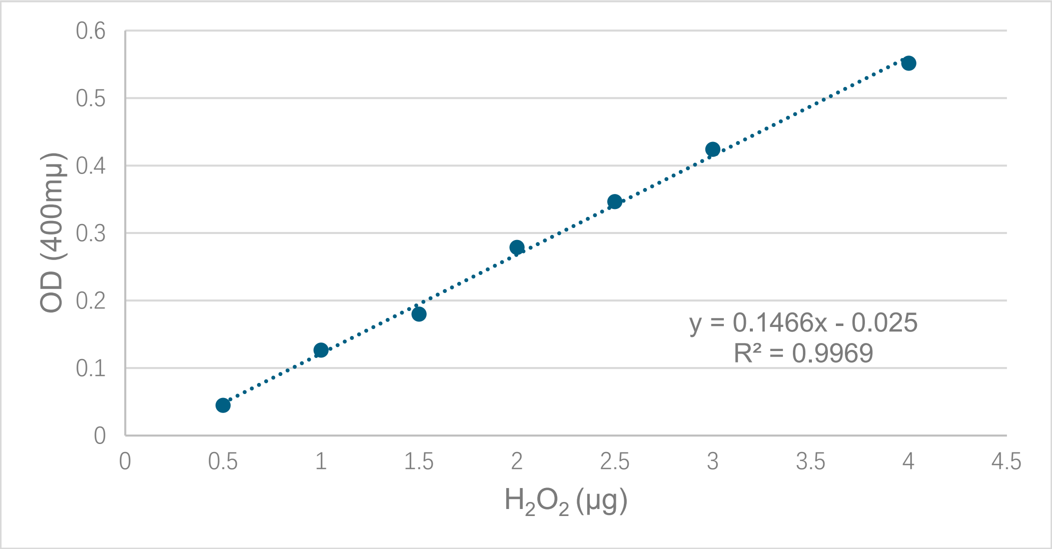

Figure S2 Standard curve of H_2O_2 production
